# What Is the Evidence for Inter-laminar Integration in a Prefrontal Cortical Minicolumn?

**DOI:** 10.3389/fnana.2017.00116

**Published:** 2017-12-14

**Authors:** Ioan Opris, Stephano Chang, Brian R. Noga

**Affiliations:** The Miami Project to Cure Paralysis, Department of Neurological Surgery, University of Miami Miller School of Medicine, Miami, FL, United States

**Keywords:** integration, coordination, cortical layers, cortical minicolumns, microcircuits, prefrontal cortex, executive function

## Abstract

The objective of this perspective article is to examine columnar inter-laminar integration during the executive control of behavior. The integration hypothesis posits that perceptual and behavioral signals are integrated within the prefrontal cortical inter-laminar microcircuits. Inter-laminar minicolumnar activity previously recorded from the dorsolateral prefrontal cortex (dlPFC) of nonhuman primates, trained in a visual delay match-to-sample (DMS) task, was re-assessed from an integrative perspective. Biomorphic multielectrode arrays (MEAs) played a unique role in the *in vivo* recording of columnar cell firing in the dlPFC layers 2/3 and 5/6. Several integrative aspects stem from these experiments: 1. Functional integration of perceptual and behavioral signals across cortical layers during executive control. The integrative effect of dlPFC minicolumns was shown by: (i) increased correlated firing on correct vs. error trials; (ii) decreased correlated firing when the number of non-matching images increased; and (iii) similar spatial firing preference across cortical-striatal cells during spatial-trials, and less on object-trials. 2. Causal relations to integration of cognitive signals by the minicolumnar turbo-engines. The inter-laminar integration between the perceptual and executive circuits was facilitated by stimulating the infra-granular layers with firing patterns obtained from supra-granular layers that enhanced spatial preference of percent correct performance on spatial trials. 3. Integration across hierarchical levels of the brain. The integration of intention signals (visual spatial, direction) with movement preparation (timing, velocity) in striatum and with the motor command and posture in midbrain is also discussed. These findings provide evidence for inter-laminar integration of executive control signals within brain’s prefrontal cortical microcircuits.

## Introduction

Neural integration can be defined as the summation of excitatory and inhibitory synaptic inputs, which governs the generation of an action potential (Arnold et al., 2004). Integration of various neuronal signals within and between prefrontal cortical microcircuits plays a crucial role in cognition, perception and action (Penfield, 1958; Miller and Cohen, 2001; Opris et al., 2013; Bastos et al., 2015). Voluntary action is based on the intention to achieve a goal, and this goal determines how planning and subsequent actions lead to its achievement. However, recent studies point to evidence for neural integration of voluntary action in a prefrontal cortical minicolumn (Opris et al., 2011, 2012b, 2013; Bastos et al., 2015).

The prefrontal cortex may be regarded as an assembly of interconnected neuronal cells that sends and receives projections to/from virtually all cortical areas (sensory, motor and association), as well as to/from multiple subcortical structures (Miller and Cohen, 2001). As pointed out by Vernon Mountcastle, it is critical to identify the arrays of inputs and outputs (Mountcastle, 1997), in order to understand the integrative role of the prefrontal cortex (Miller and Cohen, 2001) and its functional organization (Kritzer and Goldman-Rakic, 1995).

Functional specialization of the brain employs cortical layers that differ from three to six layers (Shepherd, 2011). The sixth layer of cortex is, in fact, an adaptation observed exclusively in mammals, although it shares some commonalities with other species (Aboitiz, 2011; DeFelipe, 2011; Bosman and Aboitiz, 2015). A complementary role in the expression of brain function is played by the cortical minicolumns that interconnects the layers and forms functional microcircuits (Mountcastle, 1957, 1997; Shepherd and Grillner, 2010; DeFelipe et al., 2012). Just imagine if the supra-granular and infra-granular laminae would be disconnected, across the entire brain, then, one interface of the brain will be sensing without perceiving (i.e., being able to interpret their meaning) and the other interface will act/move randomly, without having a goal.

In the prefrontal cortex, the vertical “chains” of neurons called minicolumns (Mountcastle, 1957, 1997; Buxhoeveden and Casanova, 2002) are surrounded by inhibitory cells, forming a curtain of inhibition (Szentágothai and Arbib, 1975; see the Schematic diagram). Cortical neurons within prefrontal minicolumns are inter-connected across all layers: three upper layers (L1–L3), one granular layer (L4) and two lower layers (L5/L6). The granular layer receives the “sensory inputs” via the thalamus (Constantinople and Bruno, 2013). According to the concept of “three-stratum” functional module, lower layers execute the associative computations elaborated in upper layers (Buxhoeveden and Casanova, 2002; Casanova et al., 2011; Opris, 2013). The upper layers consist of small pyramidal cells that form vertical connections with the larger pyramidal neurons of the lower layers that generate most of the output from the cerebral cortex to other cortical/subcortical parts of the brain (Buxhoeveden and Casanova, 2002; Gabbott et al., 2003, 2005). Cortical microcircuit modules are forming topographic maps in visual and motor cortices (Mountcastle, 1997; Thomson and Lamy, 2007; Kaas, 2012), as well as in auditory (Allen et al., 2017), somatic (Mountcastle, 1997), taste (Peng et al., 2015) and smell (Qu et al., 2016) modalities of these sensory cortices. A modular network can be partitioned into nodes that are “densely interconnected internally” but only sparsely to other subsets (Chung et al., 2016).

The aim of this perspective is to examine the integration of perceptual and behavioral signals in the inter-laminar prefrontal cortical microcircuits (Opris et al., 2011, 2012a,b, 2013; Opris and Casanova, 2014). Given that the prefrontal cortex is the “seat” of the highest brain functions (Fuster, 2001), understanding the site of integration and the functional role of this computational mechanism is essential.

### Cortical Layers and Functional Integration

Let’s begin with few questions that address the core of modular structure and its functional role.

#### Why Have Cortical Layers?

Cortical layers serve multiple purposes: some serve more local microcircuits while others act as interconnecting loops (thalamo-cortical) or large-scale networks (bottom-up) that have a multifunctional role.

**Schematic diagram:**
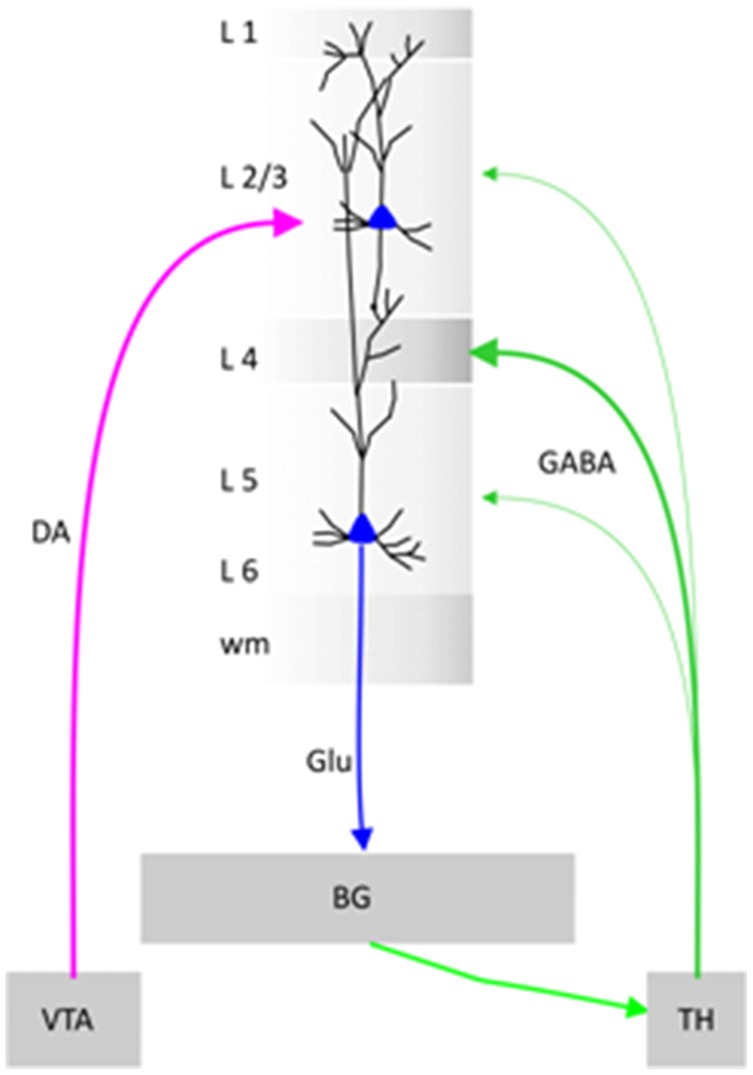
Illustration of a cortical minicolumn that integrates information from Ventral Tegmental Area (VTA) and Thalamus (TH). BG stands for Basal Ganglia, DA for Dopamine, Glu for Glutamate and GABA for gamma butyric acid.

#### What Is the Function of Layering?

Being part of the brain’s connectome (Hilgetag et al., 2016) including the entirety of brain’s connections, cortical layers serve local microcircuits processing sensory or motor information, as well as the interconnected loops (thalamo-cortical) or large-scale networks: (i) bottom-up, sensory-to-motor; (ii) top-down, cognitive-to-motor; and (iii) inter-hemispheric, for coordination of various behaviors, serving a multifunctional purpose (Miller and Phelps, 2010; Makarova et al., 2011; Georgopoulos, 2015).

#### Do Neurons in Cortex Integrate Information Across Different Layers or Across Columns?

Indeed, neurons from different cortical layers/columns communicate to each other via synchronized firing (Romo et al., 2003; Opris et al., 2013) across cortical layers, quantified by cross-correlation histograms (CCHs). Also, neuronal integration has been studied using “noise and signal correlations” or LFP-spike interactions (Bosman and Aboitiz, 2015).

#### What Is the Evidence for Integration of Information Across Several Layers?

**Figure 1:**
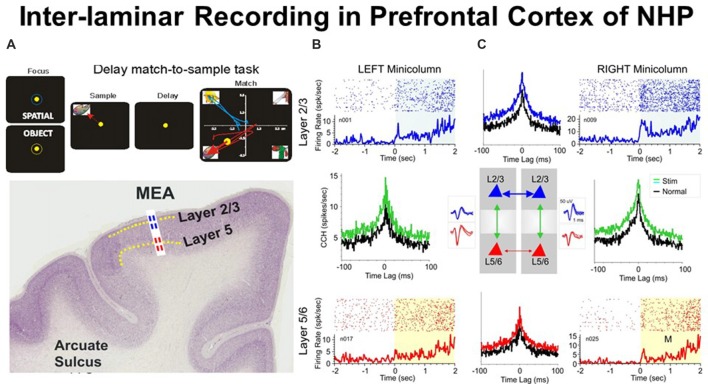
**(A)** Behavioral paradigm showing the sequence of events in the rule-based delay match-to-sample (DMS) task: (1) presentation of a “focus” image (blue or yellow ring) to initiate an “object” or “spatial” trial, respectively, and prompting cursor placement to lead to (2) presentation of the “sample” image, followed by cursor movement into the image as the “sample” response followed by (3) a variable delay period of 1–60 s with a blank screen, followed by (4) the “match” phase in which the “sample” image was presented along with 1–6 other nonmatch (distractor) images on the same screen. Cursor movement into the correct (match target) image for ≥0.5 s produced a juice reward via a sipper tube mounted next to the animal’s mouth. Placement of the cursor into a nonmatch image for ≥0.5 s caused the screen to blank without reward delivery. Intertrial interval: 10.0 s. **(B)** Illustrated coronal section in rhesus monkey brain showing relative location of supra-granular L2/3 (blue) and infra-granular L5 (red) with tract used for placement of conformal multielectrode array (MEA) recording probe. **(C)** Interlaminar activity recorded from adjacent prefrontal minicolumns during DMS task performance. Recording array: center insert shows the conformal MEA positioned for simultaneous interlaminar–columnar recording from adjacent mini-columns left and right with corresponding L2/3 and L5 cell pair waveforms (blue and red). Individual trial rasters and average peri-event histograms (PEHs) obtained from two cell pairs recorded simultaneously from L2/3 (blue) and L5 (red) in minicolumn format over ± 2.0 s relative to match phase (**A**) onset (0.0 s) in a single DMS session. Cross-correlation histograms (CCHs) for the same cell pairs in each minicolumn are shown (between raster-PEH displays) in left and right mini-columns for Pre (black, −2.0 s to 0.0 s) and Post (green, 0.0 s to +2.0 s) time intervals relative to match phase onset (M, 0.0 s). CCHs show increased inter-laminar synchronization (larger correlation peaks) for both cell pairs during target selection in the match phase (green, post) relative to similar correlations between the same cell pairs constructed before phase onset (pre, −2.0 s to 0.0 s). With permission from Opris et al. (2012b).

As seen in Figure 1, the neurons in prefrontal cortical layers L2/3 and L5 change firing patterns during the presentation of the matching target (together with the non-matching distractors), resulting in inter-laminar CCHs changes (increases the number of coincident spikes) that may represent trans-laminar integration of information (Foxworthy et al., 2013; Opris et al., 2013). Information flows through cortical layers in a feed-forward manner (Bastos et al., 2015), going from layer 4, to layers 2/3 and onwards (Bastos et al., 2012). Another view maintains that cortical layers can have distinct inputs that activate them, triggering spikes when the integration input bypasses a threshold (Opris and Casanova, 2014).

#### What Is the Hypothesis of Prefrontal Cortical Inter-laminar Integration?

The integrative hypothesis posits that integration of perceptual signals from supra-granular layers with the behavioral signals in the infra-granular layers takes place in the prefrontal cortical inter-laminar microcircuits (Opris et al., 2011, 2012a,b, 2013; Takeuchi et al., 2011).

#### What Is the Function of Each Prefrontal Cortical Layer?

Layer 1: Cross-modality modulation is achieved through layer L1 cell-mediated inhibitory and disinhibitory circuits (Ibrahim et al., 2016). Neurons in layer L1 form “canonical neuronal circuits” to control information processes in both supra- and infra-granular cortical layers (Lee et al., 2015). Projections of neurons to layer L1 from all cortical inputs has been shown to make a significant contribution to the integration process throughout the neocortex (Mitchell and Cauller, 2001).

Layer 2/3: This layer provides the integration of perceptual stimuli by inter-areal bottom up connections and the integration of perception with action by trans-laminar connections and top-down cortico-subcortical connections (Opris et al., 2011, 2012a,b, 2013).

Layer 3: This layer provides inter-hemispheric callosal connections (Georgopoulos, 2015).

Layer 4: The primary visual cortex receives orientation- and direction-tuned inputs from thalamus in layer 4 (Sun et al., 2016).

Layer 5: Pyramidal neurons in this layer integrate inputs from many sources and distribute outputs to cortical and subcortical structures (Kim et al., 2015). Cortical-subcortical neurons project to subcortical structures in the thalamo-cortical loop, integrate information and generate responses more relevant to movement control, while cortico-cortical neurons are more important in visual perception.

Layer 6: This layer projects to the thalamus (Opris and Casanova, 2014).

### What Is the Evidence for Cortical Inter-laminar Integration?

#### Evidence for Functional Integration in Cortical Minicolumns

It is obvious that cortical neurons that interconnect to each other across layers play an important role in the emergence of brain function. To demonstrate this we show in Figure 1 an ideal example that depicts simultaneously recorded cells with differential firing in adjacent minicolums and inter-laminar layers during the match epoch of the DMS task (Figure 1A). Functional integration may be pursued based on: (i) inter-laminar; and/or (ii) inter-columnar neuronal interactions. The cross-correlograms (Figure 1C) that quantify these interactions between cells provide crucial evidence for the integration process (Opris et al., 2012b).

#### Columnar Inter-laminar Processing in Prefrontal Cortex during “Target Selection”

Figure 1C shows an example of inter-laminar interaction between two cell pairs with individual firing rates displayed in raster/peri-event histograms (PEHs) during the epoch between matching image presentation (match phase onset) and offset of the matching “target” (0.0 s ± 2.0 s). Cell pairs in this study (Opris et al., 2012b) were recorded on “appropriate sets of adjacent pads” (corresponding to Minicolumns 1 and 2) on the biomorphic multielectrode arrays (MEAs), shown in the illustration of both trans-laminar cell pairs in L2/3 and L5/6 (Figure 1C). Neurons in both, upper and lower layers showed “significant increases” in mean firing rate (*p* < 0.001) in the “post”-match phase (0.0 s to 2.0 s) associated with target selection (Figure 1A) and the decision making phase of the task (Opris et al., 2012b), relative to the “pre”-match phase (−2.0 s to 0.0 s). An important observation highlighted with this type of recording configuration was that layer L2/3 neurons in the same minicolumn, exhibited higher firing rates in the “post”-match epoch (0.0 s to +2.0 s; Figure 1C, upper raster/PEHs) than neurons in L5 of the same, over the same time period (Figure 1C, lower raster/PEHs).

Precise “functional connections” between single units (cells) within each minicolumn were provided by cross-correlation histograms (CCHs), represented for individual L2/3 and L5 cell pairs recorded on vertically positioned electrode pads (Figure 1B) of the MEA (Opris et al., 2011, 2012b, 2013; Takeuchi et al., 2011). Normalized synchronized firing (shown by CCHs) for both minicolumns were shown in Figure 1C for cell firing in the displayed PEHs: (i) “pre”-match epoch (−2.0 s to 0.0 s, Pre, black curve); or (ii) “post”-match epoch (0.0 s to +2.0 s, Post, green) for the same cell pairs. Although both CCHs depict a “significantly correlated firing” (*p* < 0.001; *t*-test), the differences in max correlation for both neuron pairs suggest that inter-laminar firing was more synchronized in the “post”-match epoch (0.0–2.0 s) than in the “pre”-match epoch (*p* < 0.001; *t*-test).

#### Laminar Inter-columnar Processing in Prefrontal Cortex during “Target Selection”

The demonstration of functional connections between individual cells within same cortical layer and different minicolumns was provided by CCHs (Opris et al., 2012a) plotted for individual L2/3-vs.-L2/3 and L5-vs.-L5 cell pairs, recorded on horizontally positioned electrode pads on the MEA. Normalized correlograms for cell pairs are presented in Figure 1C. Although both correlograms (L2/3-vs.-L2/3 and L5-vs.-L5) show significantly correlated firing (Post vs. Pre: *p* < 0.001), the differences in peak correlation for both cell pairs indicate that intra-laminar inter-columnar firing was more synchronized in layer 2/3 than in layer 5 (*p* < 0.001). This supports the idea of laminar integration in supra-granular layers (Petro and Muckli, 2017).

#### Cortical-Subcortical Interaction

Cortical-subcortical integration of sensory and motor signals occurs during cortico-striatal interactions funneling signals in the cortico-thalamic loops (McFarland and Haber, 2002). Two recent results reported by Opris et al. (2013) and Santos et al. (2014) have demonstrated differential (pre)frontal cortico-striatal interaction during the planning and preparation for movement in monkeys.

#### Laminar Inter-hemispheric Processing in Prefrontal Cortex in Layer 3

Neurons in Layer 3 of fronto-parietal cortices provide important interhemispheric callosal connections necessary for the coordination of movement (Georgopoulos, 2015). Obviously, inter-hemispheric integration is playing a key role such as coordination.

#### Comparison of Inter-laminar Integration in the Correct vs. Error Trials

**Figure 2:**
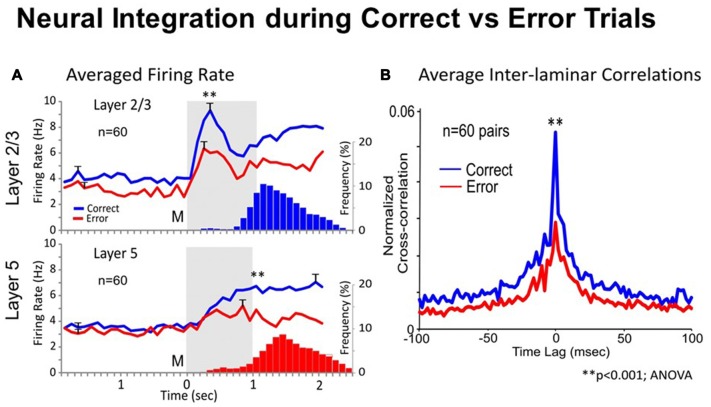
Neural integration during correct vs. error trials. **(A)** Average Firing Rate. Mean PEHs during match phase averaged over all recorded interlaminar prefrontal cortex (PFC) cell pairs (*n* = 60), L2/3 (upper) and L5 (lower), on correct (blue) vs. error trials (red) summed across animals and sessions with ≥2 cell pairs recorded in same behavioral session from the same MEA. Blue and red histograms (bars) below PEHs show the associated mean frequency distributions of match response latencies (s) for correct (upper) and error (lower) trials plotted on the same time-base as the PEHs relative to match phase onset (0.0 s). **(B)** Average Inter-laminar Correlation. Mean CCHs for the same inter-laminar cell pairs (*n* = 60) constructed from correct (blue) and error (red) trials. ANOVA, *F*_(1,119)_ = 14.18, **p < 0.001, difference in peak mean correlation.

One way to test whether trans-laminar integration takes place in cortical minicolumns is to compare the inter-laminar firing in correct vs. error trials. A trial was considered correct when the animal responded adequately to each instruction of the DMS task’s event sequence (shown in Figure 1A), receiving a drop of juice as reward, or an error trial, when the animal failed to obey one or more instructions, and was not rewarded (Opris et al., 2012b). Experiments by Opris et al. (2012b) compared correlated firing of pairs of cells from layers L2/3 and L5 during the match epoch (dealing with target selection) under correct vs. error trials. Figure 2A shows that cell pairs (in layers L2/3 and L5) that exhibited increased firing during the match epoch in correct trials (left), reduced their firing on error trials, with the inappropriate target being selected (right). The trend of increased (respectively decreased) correlated firing in correct (respectively error) trials was present across entire subpopulation of prefrontal cell pairs, as shown in Figure 2A. Moreover, the significant increase (decrease) in the mean CCH peaks (*p* < 0.001), provides evidence for the enhanced (lack of) inter-laminar correlated firing between L2/3 and L5 in correct (error) trials, as shown in Figure 2B. Taken together, these unique simultaneous recordings in prefrontal cortical layers (and minicolumns) of rhesus macaques, during *in vivo* performance of a cognitive task, provide evidence for prefrontal cortical inter-laminar integration during correct trials and lack of integration during error trials.

#### Integration of Spatial Perception with Action

**Figure 3:**
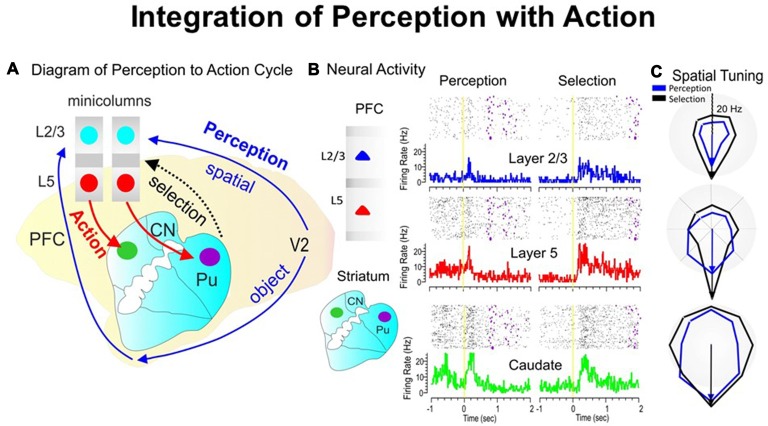
The perception-to-action cycle with the behavioral paradigm. **(A)** The illustration of the perception-to-action cycle. The diagram depicts the flow of spatial and object signals during perceptual and executive selection of target stimuli in a rhesus macaque brain. In visual cortical area V2, visual information splits into dorsal (spatial signals) and ventral (object signals) pathways to the top of executive hierarchy in the PFC, and then top-down through the cortico-striatal-thalamo-cortical loops. Blue arrows depict the perceptual flow of information while red arrows indicate the action (executive) signal flow from prefrontal cortical layer 5 to the dorsal striatum, with the red dotted arrow indicating the thalamo-cortical projection in the cortico-striatal-thalamo-cortical loop. The two adjacent cortical minicolumns with red and blue filled circles indicate inter-laminar simultaneous recordings, while caudate-putamen recording are shown in green and pink circles. **(B)** Example of simultaneous individual activity (individual trial rasters and peri-event histograms) of single neurons recorded in prefrontal cortical layers L2/3 (blue) and L5 (red) with the conformal MEA and caudate n. (green) during “sample” (left part) and “match” target presentation (right part) on spatial trials during a single session (*n* = 120 trials). The purple marks in the rasters represent the time when the target was reached. **(C)** Directional tuning plots (blue for perception and black for executive selection) depict firing preference, measured by the radial eccentricity (in spikes/s or Hz) in the polygonal contour for the eight different target locations on the screen where images appear. The overlay tuning plots compare firing preferences on spatial trials for the same cells during “sample” (perception) and “match” (selection) presentation. The tuning vectors also show the magnitude of firing for preferred locations during the encoding (left panel) and selection (right panel) phases of the task on spatial trials. Spatial trials tuning vectors (black) show the same preferred directionality (i.e., 270°) during the encoding and selection phases in both PFC layers and in caudate nucleus, suggesting parallel processing streams/loops through cortical minicolumns and striatum and likely through the entire thalamo-cortical loop. The radius of polar plots is represented in Hz and tuning amplitude is measured in Hz, as well. Asterisks: **p < 0.001, ANOVA.

During the perception-to-action cycle, when the brain plans a movement, the executive mechanism coordinates the interactions between the environment and the perceptual/sensory-motor/executive systems (Fuster, 2000; Opris et al., 2011, 2013). On top of the executive hierarchy, the prefrontal microcircuits are assumed to “bind perceptual and executive control” functional signals to coordinate goal-driven behavior (Figure 3A). Here, we highlight the recorded neuronal “firing simultaneously in prefrontal cortical layers and the caudate-putamen” of rhesus monkeys (see Figure 3B), trained in a spatial-vs.-object version of the visual match-to-sample task (Opris et al., 2013). During the perception and executive selection epochs, cell firing in the prefrontal layers and caudate-putamen exhibited “preferences for the same location” on spatial-trials, but not on object-trials.

#### Transformation of Perceptual Signals into Action

When the perceptual-executive circuit is activated by the stimulation of prefrontal infra-granular-layers with electrical stimuli “patterns” obtained from supra-granular-layers, Opris et al. (2013) could replicate spatial preferences (similar to neural tuning) in percent correct performance, on spatial trials (Figure 3C). These results show that inter-laminar prefrontal microcircuits play causal roles in the perception-to-action cycle (Mahan and Georgopoulos, 2013; Opris et al., 2013).

#### Does the Number of Distractor Images Affect the Inter-laminar Integration?

**Figure 4:**
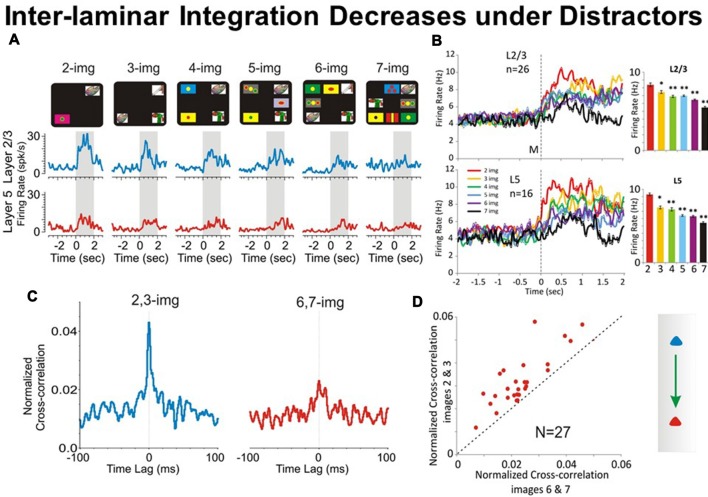
Effect of number of images on PFC columnar firing. **(A)** Example PEHs comparing neuron firing in PFC layers L2/3 (blue) and L5 (red) as a function of the number of images presented (upper: display screens) in the match phase on Object type trials in the DMS task. **(B)** Population PEHs depicting the activity of prefrontal cells from layers L2/3 (*n* = 16) and L5 (*n* = 26) on all types of trials with different numbers of images (2, 3, 4, 5, 6 and 7) presented during match phase in the DMS task (L2/3: *F*_(6,1039)_ = 8.29, *p* < 0.001; L5: *F*_(6,639)_ = 8.64; *p* < 0.001, ANOVA). **(C)** Example inter-laminar CCHs for trials with a few (2 and 3 images) vs. many (6 and 7 images) distracter images constructed from the same interlaminar L2/3-L5 cell pair shown in **(A)**. **(D)** Normalized population CCHs for trials with low (2, 3 red) vs. high (6, 7 blue) numbers of images in the match phase consisting of the average correlation coefficients across individual CCHs from 27 different inter-laminar cell pairs. Scatter plot showing differential distributions of individual CCH peak correlation coefficients on trials with low vs. high numbers of images for the same cell pairs (*n* = 27) comprising the population CCH. **p < 0.001, *p < 0.01, ANOVA.

In the DMS task in Figure 1A the distractor images are those images that represent a different object than the sample image. As shown previously (Opris et al., 2011, 2012a,b, 2013), a major factor influencing the selection of the matching target in the match phase of the DMS task was the number of distractor images presented with the “sample” image in a given trial (see Figure 4). The increase in task difficulty via increasing the number of distractors, allowed the animal to make a sizeable amount of error trials for comparison to the correct trials (Opris et al., 2012a). In Figure 4A is shown a gradual “decrease in cell pair firing in layers L2/3 and L5” as a function of the number of images presented during the matching phase. Consistent with our prior results (Opris et al., 2012a,b), neuronal population mean firing rates in layers L2/3 and L5 (Figure 4B) confirmed this decrease as a function of the number of distractors in the match phase (*p* < 0.001). Moreover, this decrease was also expressed in terms of “correlated firing” between L2/3-L5 cell pairs, as shown in Figures 4C,D, in which CCHs on trials with few (2 or 3) distracter images exhibited significantly higher correlations than on trials with more (6 or 7) distracter images (*p* < 0.01). The decrease in inter-laminar correlated firing is consistent with a decrease in integration caused by the increase in the number of distractor targets. Likely the decrease in task performance is due to an increase in the “cognitive work load” of the task (Kelley and Lavie, 2011; Opris et al., 2011).

#### Causal Relations to Columnar Integration

**Figure 5:**
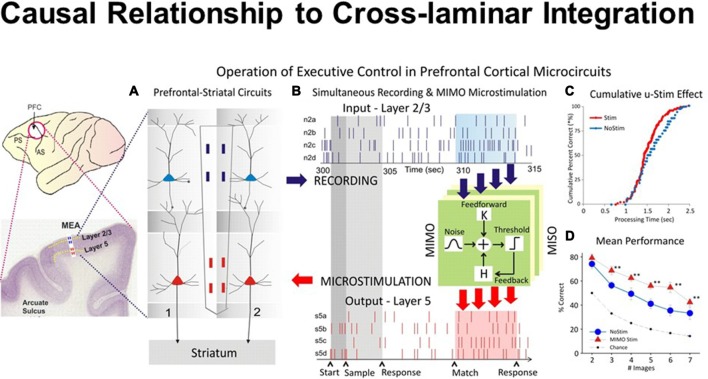
Closing inter-laminar loops in PFC with multi-input multi-output (MIMO) model generated stimulation. **(A)** Diagram of the interfacing of MIMO model with conformal MEAs between L2/3 and L5 during task performance. Electrical stimulation delivered to MEA pads in L5 via patterns of pulses (biphasic) recorded and derived from the same L5 locations on successful trials by the MIMO model. **(B)** Firing of L2/3 and L5 located columnar neurons as shown in Figure 1C recorded on line and fed to MIMO model shown in **(A)**. Shaded areas indicate time of match response execution during DMS trial, and the illustrated firing in L5 which is the same pattern as the delivered stimulation on trials with inappropriate L2/3 firing. **(C)** Changes in cumulative response latencies (processing time) from match phase onset (“0”) during trials with stimulation delivered in the manner shown in **(A,B,D)**. Increase in performance across trials with increasing difficulty as a function of the number of match phase distracter images on trials that received MIMO stimulation in the manner shown in **(A)**. **(D)** Differential effects of MIMO stimulation on spatial vs. object trials showing more enhancement on spatial trials ranging in delays of 1–20 s. **p < 0.001, ANOVA.

To test causal relationship to columnar inter-laminar integration, the recorded firing of neurons was examined via a nonlinear multi-input multi-output (MIMO) model, which extracted and characterized multi-laminar firing patterns during performance in the DMS task (Hampson et al., 2012). Figure 5 provides causal evidence for the integration of natural signal flowing through the PFC microcircuits with the injected signal via stimulation. Minicolumnar neuronal firing of the PFC neurons was recorded from rhesus macaques trained in a DMS task, via biomorphic MEAs that provided signals from neurons in prefrontal cortical layers 2/3 and 5 (Opris et al., 2015a). The MIMO device sent patterns of electrical stimuli via *stimulation* to prefrontal cortical layer 5, during columnar “inter-laminar integration” at the time of target selection (Opris, 2013). Such stimulation improved (augmented) normal task performance significantly (Figure 5D). The executive control circuit was facilitated by applying stimulation patterns in the prefrontal cortical infra-granular-layers similar to the patterns coming from the supra-granular-layers that produced enhanced spatial preference in performance percentage, similar to neural tuning. Thus, inter-laminar prefrontal microcircuits may play causative roles in the cognitive perception-to-action cycle (Hampson et al., 2012; Opris, 2013). Such causal relations to integration of cognitive signals provide direct evidence that cortical minicolumns may represent the turbo-engines of the brain (Jones, 2000; Jones and Rakic, 2010; Opris et al., 2013; Chapman and Mudar, 2014).

#### Integration across Hierarchical Levels of Processing

**Figure 6:**
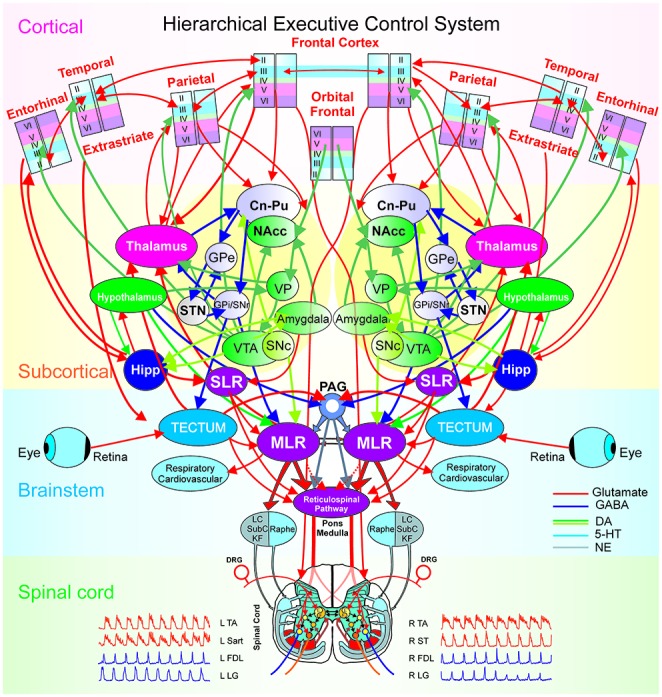
The hierarchical executive control system for movement. The executive control system is anatomically organized via a hierarchical architecture of cortical modules (layers, minicolumns with microcircuits), subcortical nuclei (basal ganglia and thalamus with cortical-subcortical-thalamic loops; hippocampus and hypothalamus), brainstem (midbrain, pons, medulla with cortical-brainstem networks), and spinal cord (locomotor central pattern generators, CPG). At the higher level in the hierarchy are the columnar laminar modules of frontal (cognitive), parietal (motor), extrastriate (visual), temporal, orbital frontal cortices (emotion) and entorhinal (limbic), Beneath, are the subcortical structures: striatum (caudate, Cn, putamen, Pu and nucleus accumbens, NAcc), globus pallidus [GPe/GPi (external/internal segments), the dopaminergic ventral tegmental area (VTA), the ventral pallidum (VP), substantia nigra (SNc/SNr: pars compacta/pars reticulata), subthalamic nucleus (STN) and the thalamus. Motivation and emotion are processed by the hypothalamus and amygdala, respectively. The coordination of the navigation systems involves the frontal cortex and the hippocampus (Hipp). The subthalamic locomotor region (SLR) is a subcortical center for coordinating locomotion. At the level of the brain-stem and spinal cord, locomotion is initiated by the direct activation or disinhibition of the mesencephalic locomotor region (MLR) and/or the reticulospinal (RS) pathway. Stimulation of the MLR activates reticulospinal neurons which project through the ventrolateral funiculus to activate spinal locomotor central pattern generator neurons, in part, by the release of excitatory amino acids. The reticulospinal pathway is considered to comprise the primary “command pathway” for the initiation of locomotion. MLR stimulation also activates in parallel, multiple monoaminergic descending pathways during centrally-generated locomotion. The flexor (F) and extensor (E) components of the locomotor CPG are activated/modulated by descending bilateral reticulospinal and monoaminergic projections as well as by crossed excitatory (▸) and inhibitory (•) segmental projections from the CPG opposite to it. Sensory afferents from skin and muscles innervate spinal neurons in the dorsal horn, intermediate zone and ventral horn to fine-tune the locomotor step cycle. Details of the flexor and extensor components of the CPG are omitted in order to emphasize general interconnections between them and their target neurons. LC, locus ceruleus; SubC, subceruleus; KF, Kölliker-Fuse; DRG, dorsal root ganglia; L, left; R, right; TA, tibialis anterior; Sart, Sartorius; FDL, flexor digitorum longus; LG, lateral gastrocnemius; ST, semitendinosus.

Figure 6 illustrates modular integration across hierarchical levels. Executive control has been defined as the brain’s ability “to control thought and action” by coordinating “multiple systems and mechanisms across multiple brain areas” to pursue a goal (Miller and Phelps, 2010). Examples of executive control functions include: attention, working memory, decision making, intention or motor plan and behavioral inhibition (Fuster and Bressler, 2012). Thus, the ability of the brain to exercise executive control over behavior relies upon the integration of the multiple microcircuitries (sensory, motor, reward), loops (thalamo-cortical) and large scale networks (bottom-up or top-down) with distributive encoding organized in a hierarchical manner.

The hierarchy of brain functions was introduced by Joaquin Fuster based on Hughlings Jackson’s assumption that the neocortex is the climax of the nervous system and it controls (activates and/or inhibits) the functions of lower levels. However, cortical disease led to two sets of findings: “negative” signs and symptoms from “loss of the controlling cortex” and “positive” ones from the “emergence of the lower center” (Fuster, 1990; York and Steinberg, 2011). This implied an anatomical and physiological hierarchy of centers within the brain, with higher ones activating or suppressing the function of lower ones (Fuster, 1990; York and Steinberg, 2011).

Our focus is on the hierarchy of neural circuitry underlying the executive control of behavior (Figure 6) spanning from the frontal and parietal cortices, subcortical structures like the basal ganglia and thalamus, the brainstem and the spinal cord. The hierarchical integration of various stimuli (Hirabayashi et al., 2013a,b) in the executive function, may follow a bottom-up integration of visual information (Felleman and Van Essen, 1991) while the coordination of movement kinematics is performed in a top-down manner according to a prior intention/plan (Noga and Opris, 2017). At the top of the executive hierarchy are the frontal (premotor) and parietal (motor) cortical microcircuits, interconnected within thalamo-cortical loops through cortico-striatal projections and further in the brainstem to the mesencephalic locomotor region (MLR) and the central pattern generators in the spinal cord (Noga and Opris, 2017).

## Future Directions

The following research topics deserve attention in the future neuroscience research. The first topic should be on the prefrontal cortical interactions with cortical (parietal, temporal and occipital) and subcortical (basal ganglia, thalamus, hypothalamus, hippocampus, amygdala) brain regions (Opris et al., 2013) during emergence of behavior and brain functions. The next topic may deal with the organization of prefrontal cortical connections within the brain’s connectome from microcircuits to large scale networks (Hill et al., 2012; Markov et al., 2013; Markram et al., 2015; Reimann et al., 2017). The brain’s connectome needs to be dissected at the microcircuit level. More evidence is needed for the functional organization across the prefrontal cortical areas that form a hub in the brain’s connectome (Sato et al., 2016). Finally, evidence is needed for understanding brain disorders like autism, schizophrenia, Alzheimer’s disease, attention deficit disorder or drug addiction (Opris and Casanova, 2014; Opris et al., 2015b).

## Conclusion

The findings discussed here provide a unique insight into the inter-laminar integration of neural signals and illustrate the role of prefrontal cortical microcircuits in the executive control of behavior in the primate brain. This would have been impossible without the use of biomorphic multielectrode array that was tailored specifically for recording the columnar-laminar micro-architecture of the neocortex, in behaving monkeys during a cognitive task. The executive control role of prefrontal minicolumns was demonstrated by spatial preference signals, integrated and transformed into action signals which coded the intention to move to the same spatial location (Opris et al., 2013). A causal relationship using signals recorded in the upper prefrontal layers and feeding a similar pattern of electrically stimuli in the lower layers demonstrated the causal role of prefrontal mincolumn as a cognitive “turbo-engine. Prefrontal cortical integration of perceptual signals (in supra-granular cortical layers), together with the behavioral (infra-granular layers) and reward signals (midbrain) results in the emergence of various brain functions. These cortical modules and their microcircuits represent the building blocks of these brain functions, with information passing through them in a hierarchical manner: from sensory to cognitive (bottom-up) structures in the prefrontal cortex and top-down to motor structures processing action and behavior, via thalamo-cortical loops.

## Author Contributions

This manuscript was written by IO together with SC and BRN.

## Conflict of Interest Statement

The authors declare that the research was conducted in the absence of any commercial or financial relationships that could be construed as a potential conflict of interest.
